# Serum chloride as a respiratory failure marker in amyotrophic lateral sclerosis

**DOI:** 10.3389/fnagi.2023.1188827

**Published:** 2023-05-24

**Authors:** Umberto Manera, Maurizio Grassano, Enrico Matteoni, Alessandro Bombaci, Rosario Vasta, Francesca Palumbo, Maria Claudia Torrieri, Paolo Cugnasco, Cristina Moglia, Antonio Canosa, Adriano Chiò, Andrea Calvo

**Affiliations:** ^1^“Rita Levi Montalcini” Department of Neuroscience, University of Turin, Turin, Italy; ^2^SC Neurologia 1U, AOU Città della Salute e della Scienza di Torino, Turin, Italy; ^3^Neurology Unit, Department of Clinical and Biological Sciences, San Luigi Gonzaga Hospital, University of Turin, Orbassano, Italy; ^4^Institute of Cognitive Sciences and Technologies, Rome, Italy

**Keywords:** amyotrophic lateral sclerosis, serum chloride, respiratory failure, survival, non-invasive ventilation

## Abstract

Respiratory failure is the most common cause of death in patients with amyotrophic lateral sclerosis (ALS) and occurs with great variability among patients according to different phenotypic features. Early predictors of respiratory failure in ALS are important to start non-invasive ventilation (NIV). Venous serum chloride values correlate with carbonate (HCO3-) blood levels and reflect metabolic compensation of respiratory acidosis. Despite its wide availability and low cost, few data on serum chloride as a prognostic marker exist in ALS literature. In the present study, we evaluated serum chloride values at diagnosis as prognostic biomarkers for overall survival and NIV adaptation in a retrospective center-based cohort of ALS patients. We collected all ALS patients with serum chloride assessment at diagnosis, identified through the Piemonte and Valle d’Aosta Register for ALS, evaluating the correlations among serum chloride, clinical features, and other serum biomarkers. Thereafter, time-to-event analysis was modeled to predict overall survival and NIV start. We found a significant correlation between serum chloride and inflammatory status markers, serum sodium, forced vital capacity (FVC), ALS functional rating scale-revised (ALSFRS-R) item 10 and 11, age at diagnosis, and weight loss. Time-to-event analysis confirmed both in univariate analysis and after multiple confounders’ adjustment that serum chloride value at diagnosis significantly influenced survival and time to NIV start. According to our analysis, based on a large ALS cohort, we found that serum chloride analyzed at diagnosis is a low-cost marker of impending respiratory decompensation. In our opinion, it should be added among the serum prognostic biomarkers that are able to stratify patients into different prognostic categories even when performed in the early phases of the disease.

## 1. Introduction

Amyotrophic lateral sclerosis (ALS) is the most common problem of motor neuron diseases, being invariably characterized by the progressive degeneration of both upper and lower motor neurons (Hardiman et al., 2017). Respiratory failure is the most common cause of death in ALS patients and occurs generally after a median time of 2–3 years after disease onset but with great variability among patients according to different phenotypic features (Chio et al., 2022). Early prediction of respiratory failure in ALS is of undoubted importance to start ventilation at the right time. Several respiratory tests and arterial blood gas analysis (ABGs) parameters have been evaluated, showing a different efficacy in the correct evaluation of initial respiratory dysfunction among ALS phenotypes (Heiman-Patterson et al., 2018). In recent studies, maximum inspiratory pressure (MIP), overnight pulse oximetry (OvOx; Kelly et al., 2022), serum base excess (SBE), and carbonate (HCO3-; Manera et al., 2020) have been confirmed as the earliest predictors of respiratory dysfunction, being capable of detecting subtle muscle weakness and nocturnal hypoventilation.

Venous serum chloride reflects the degree of respiratory acidosis and correlates with carbonate (HCO3-) blood levels: respiratory acidosis pushes the renal proximal tubule to increase secretion of hydrogen ions, resulting in higher retention of sodium bicarbonate more than sodium chloride. Although this compensatory mechanism helps in ameliorating acidosis, the final result is compensatory metabolic alkalosis and decreased serum chloride concentrations.

Despite its wide availability and low cost, also considering evidence that comes from other chronic pulmonary conditions (Naal et al., 2018), few data on serum chloride as a prognostic marker exist in literature. Some studies, evaluating ALS clinical trial data (Stambler et al., 1998) and a prospective ALS patient cohort (Hadjikoutis, 2001), observed that serum chloride strictly correlates with prognosis and respiratory symptom development. However, in multidisciplinary ALS clinics, serum chloride is barely considered as a marker of impending respiratory failure (Melo et al., 1999), and many of the most recent studies on early predictors of respiratory decline did not even mention it (Michels et al., 2022).

In the present study, we evaluated serum chloride values at diagnosis as a prognostic biomarker for overall survival and non-invasive ventilation (NIV) adaptation in a retrospective center-based cohort of ALS patients.

## 2. Methods

### 2.1. Study population

We collected all ALS patients with serum chloride assessment at diagnosis, identified through the Piemonte and Valle d’Aosta Register for ALS, and diagnosed between 1 January 2007 and 31 December 2019. All patients met the revised El Escorial diagnostic criteria for definite, probable and probable laboratory-supported ALS. We restricted analyses to individuals with available blood exam data within 3 months of ALS diagnosis. Previous and concomitant diseases were assessed, and participants with significant renal comorbidities or other medical conditions that could influence serum chloride levels were excluded. For each patient, we collected sex, date of onset, site of onset, date of diagnosis, ALSFRS-R at diagnosis, weight at diagnosis, weight before disease onset, forced vital capacity (FVC%) at diagnosis, serum chloride (mmol/L), serum sodium (mmol/L), serum potassium (mmol/L), white blood cell count (WBC, 10^9^/L), neutrophils count (10^9^/L), lymphocyte count (10^9^/L), data of NIV start, and date of death/tracheostomy from 1 January 2007 until 31 December 2021 (censoring date). The ALSFRS slope at the time of diagnosis (ΔALSFRS-R) was calculated for each patient using the following formula: (48 – ALSFRS-R score at diagnosis)/(time from onset to diagnosis). Moreover, the neutrophil-to-lymphocyte ratio (NLR) was calculated and used as a marker of inflammatory status (Leone et al., 2022).

### 2.2. Statistical analysis

Differences of discrete and continuous variables of interest were analyzed using the χ^2^-test Mann–Whitney *U*-test, as appropriate. A *p*-value <0.05 was considered to be statistically significant. Correlation analysis among serum chloride, clinical features and exams, and other serum biomarkers was performed using Pearson’s correlation coefficient. Time-to-event analysis was modeled performing backward stepwise Cox regression (unadjusted and adjusted) and Kaplan–Meier curves with the log-rank test were then performed to predict survival and NIV start. The variables included in the time-to-event analysis have already been recognized as significant prognostic factors in previous studies (Calvo et al., 2017; Westeneng et al., 2018) and showed also in our cohort a significant prognostic effect in univariate analysis.

Anonymous data were collected in an Excel (Version 14.0.4760.1000, 64 bit) dataset. Data were analyzed using IBM SPSS Statistics for Windows, version 28.0.1.0 (Armonk, NY: IBM Corp.).

### 2.3. Standard protocol approvals, registrations, and patient consents

The study design was approved by the Ethical Committee of the Azienda Ospedaliero-Universitaria Città della Salute (Prot. N. 0036344). All patients signed a written informed consent.

## 3. Results

We collected serum chloride values for 760 ALS patients at diagnosis. The descriptive statistics of the cohort are summarized in Table 1.

**Table 1:** Descriptive statistics.

	Median (IQR)
Age at onset (years)	67.6 (60.1–74.5)
Onset-diagnosis interval (months)	8.0 (5.0–13.0)
ALSFRS-R total score	43.0 (39.0–45.0)
ΔALSFRS-R at diagnosis (point loss per month)	0.600 (0.285–1.200)
Serum chloride (mmol/L)	103.0 (101.0–105.0)
Serum sodium (mmol/L)	142.0 (140.0–143.0)
Serum potassium (mmol/L)	4.2 (3.9–4.4)
Creatinine (mg/dL)	0.74 (0.61–0.89)
FVC (%) at diagnosis	92.0 (73.0–104.0)
Weight loss (kg/months)	0.27 (0.00–1.00)
Survival (months)	22.4 (11.9–43.0)
	*n* (%)
Sex	
Male	416 (54.7)
Female	344 (45.3)
Site of onset	
Bulbar onset	257 (33.8)
Upper limbs onset	212 (27.9)
Lower limbs onset	280 (36.8)
Respiratory onset	11 (1.4)
Total	760 (100.0)

Our population statistics generally reflected the median values of the general PARALS populations in terms of sex ratio, age at onset, site of onset, FVC%, and rate of progression. Median serum chloride values were 103.0 mmol/L (IQR 101.0–105.0 mmol/L), without showing any significant difference according to sex (Mann–Whitney U-test *p* = 0.936) and between patients with bulbar and spinal onset (Mann–Whitney *U*-test *p* = 0.329). Respiratory onset patients (*N* = 11) showed significantly lower serum chloride values when compared with other patients (median 98 mmol/L, IQR 94–105 mmol/L vs. median 103 mmol/L, IQR 101–105 mmol/L, *p* = 0.024).

Correlation analysis (Table 2) pointed out significant negative low-moderate correlation between serum chloride and inflammatory status markers, such as WBC count, neutrophils count, and NLR, while a positive low correlation with lymphocyte count was found. Serum sodium was moderately correlated to serum chloride, and similarly, FVC%, ALSFRS-R item 10 and 11, and survival. Age at diagnosis and weight loss was negatively correlated with serum chloride values.

**Table 2:** Correlations between serum chloride at diagnosis and other clinical features and blood biomarkers.

Serum chloride (mmol/L) vs.	Pearson correlation coefficient	*p*
WBC (10^9^/L)	−0.276	<0.001
Neutrophils (10^9^/L)	−0.328	<0.001
Lymphocyte (10^9^/L)	0.117	0.001
Neutrophil-to-lymphocyte ratio (NLR)	−0.310	<0.001
Serum sodium (mmol/L)	0.531	<0.001
Serum potassium (mmol/L)	0.088	<0.001
ALSFRS-R item 10	0.209	<0.001
ALSFRS-R item 11	0.206	<0.001
ALSFRS-R total score	0.147	<0.001
Weight loss (kg/months)	−0.101	0.009
FVC (%)	0.151	<0.001
Age at diagnosis (years)	−0.152	<0.001
Survival (months)	0.153	<0.001

Only significant correlations were showed, with Pearson correlation coefficient *p* < 0.001.

Time-to-event analysis confirmed both in univariate analysis and after multiple confounders’ adjustment that serum chloride value at diagnosis significantly influenced survival and time to NIV start (Table 3). A reduction in hazard ratio (HR) was observed by using both continuous values and after categorization: serum chloride >103.0 mmol/L determined an HR of 0.840 (IQR 0.713–0.988, *p* = 0.036) for death/tracheostomy and an HR of 0.668 (IQR 0.461–0.968, *p* = 0.033) for NIV start, after adjustment for sex, age at diagnosis, site of onset (B/S), weight loss (kg/months), ΔALSFRS-R (point loss per month), serum sodium (mmol/L), and serum potassium (mmol/L). Stratifying the Cox model by site of onset, serum chloride resulted to be highly predictive for NIV start in both bulbar onset (HR 0.850, IQR 0.766–0.942, *p* = 0.002) and spinal onset patients (HR 0.918, IQR 0.875–0.963, *p* < 0.001), while for respiratory onset patients, due to the limited number of patients (*N* = 11), the time-to-event analysis resulted in non-significance.

**Table 3:** Time-to-event analysis using Cox proportional hazard models.

Overall survival		
Serum chloride Cox unadjusted (continuous)	**0.962 (0.946–0.979)**	**<0.001**
Serum chloride Cox unadjusted (median)	**0.795 (0.682–0.926)**	**0.003**
Serum chloride Cox unadjusted (quartiles)	**0.902 (0.844–0.965)**	**0.003**
Serum chloride Cox unadjusted (quartiles categories)	1	
	0.896 (0.732–1.097)	0.289
	**0.759 (0.615–0.937)**	**0,010**
	**0.755 (0.612–0.932)**	**0.009**
Serum chloride Cox adjusted* (continuous)	**0.975 (0.957–0.993)**	**0.007**
Serum chloride Cox adjusted* (median)	**0.840 (0.713–0.988)**	**0.036**
Time to non-invasive ventilation (NIV)		
Serum chloride Cox adjusted* (continuous)	**0.951 (0.924–0.979)**	**<0.001**
Serum chloride Cox adjusted* (median)	**0.668 (0.461–0.968)**	**0.033**

Median serum chloride value was 103.0 mmol/L (IQR 101.0–105.0 mmol/L). *Adjusted for sex, age at diagnosis, site of onset (B/S), weight loss (kg/months), ΔALSFRS-R (point loss per month), serum sodium (mmol/L), and serum potassium (mmol/L). Significants are bold values (*p* >0.05).

**Figure 1:**
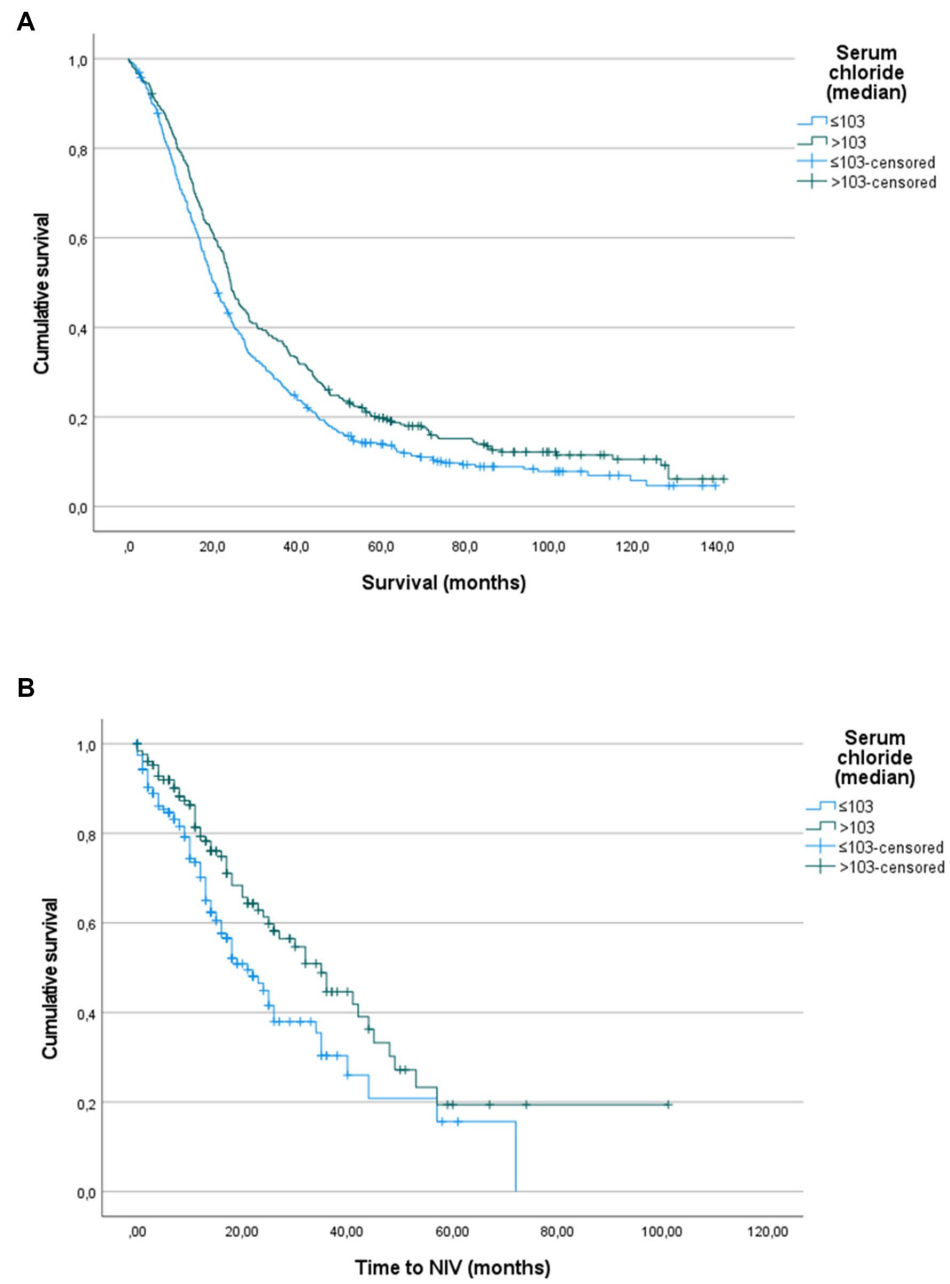
**(A)** Kaplan–Meier analysis on overall survival, performed comparing patients divided by serum chloride median value (103.0 mmol/L). Log-rank test *p* = 0.003. **(B)** Kaplan–Meier analysis on time to non-invasive ventilation (NIV) start, performed comparing patients divided by serum chloride median value (103.0 mmol/L). Log-rank test *p* = 0.010.

The Kaplan–Maier curves (Figure 1) confirmed this trend both for survival (*p* = 0.003, log-rank for pairwise comparison) and NIV indication (*p* = 0.010, log-rank for pairwise comparison).

## 4. Discussion

In this study, we confirmed with solid analysis on a large cohort that serum chloride determined at diagnosis could be used as a low-cost marker of impending respiratory decompensation in ALS patients. Its easy availability, through venous blood sampling, allows to add it among the serum prognostic biomarkers that should be performed in the early phases of the disease in order to stratify patients into different prognostic categories.

Serum chloride has already been identified as a metabolic indicator of the degree of respiratory acidosis, also in ALS patients’ cohorts, but essentially using data from a clinical trial (245 patients enrolled in the placebo group of the ALS CNTF Treatment Study Group—ACTS study; Stambler et al., 1998) or from very small prospective cohorts followed up for a short time (23 ALS patients followed up for 15 months; Hadjikoutis, 2001).

In the ACTS study, the longitudinal evaluation of serum chloride and carbon dioxide allowed to observe that mean serum chloride declined slowly at first and then dropped sharply starting at nearly 4–5 months before death (Stambler et al., 1998). In our cohort, 22.1% of patients showed serum chloride ≤100 mmol/L and 10.3% values lower than 98 mmol/L being actually definable as hypochloremic at diagnosis.

Another study (Qureshi et al., 2008), using a random effects model multivariate analysis, observed that survival was shorter in participants who had low blood chloride (HR = 0.76, *p* = 0.020) or high bicarbonate levels (HR = 1.37, *p* = 0.006). Vital capacity declined faster in individuals with lower serum chloride (*p* < 0.0001), or higher bicarbonate (*p* = 0.002). Serum chloride at the baseline affected the rate of progression of the %VC, with a decrease by 0.62 in %VC slope for each serum chloride unit decreased. Lower chloride levels were also associated in our ALS patients with a faster ALSFRS-R rate of decline.

Serum chloride was also significantly associated with systemic inflammation, measured by the increase of neutrophils and NLR values in patients with low serum chloride. This could further confirm the role of serum chloride as a marker of nocturnal hypoventilation because several recent studies found a significant association between obstructive sleep apnea syndrome, nocturnal desaturation, and some inflammatory indexes, such as the NLR (Uygur et al., 2016; Bozkuş et al., 2018).

Despite these observations, the most recent studies on blood ALS biomarkers did not include serum chloride among the evaluated candidate (Sun et al., 2020). Moreover, the majority of studies on the predictive factors of respiratory decline mainly focused on respiratory symptoms, arterial blood gas parameters, and pulmonary function tests (Michels et al., 2022). Considering both the phenotype heterogeneity and the high variability of respiratory failure expression, the use of different respiratory measures as a complementary aspect helps to better assess different aspects of respiratory function as suggested (Murray et al., 2021).

NIV is generally considered as the mainstay treatment for decreasing respiratory muscle strength and respiratory failure, affecting both quality of life (the EFNS Task Force on Diagnosis and Management of Amyotrophic Lateral Sclerosis; Bourke et al., 2006; Piepers et al., 2006; Andersen et al., 2012) and survival (Lechtzin et al., 2007). A recent survey on NIV clinical use, evidenced a substantial lack of uniformity in NIV prescription across U.S. and Europe (Heiman-Patterson et al., 2018). To the best of our knowledge, serum chloride is not considered as a respiratory biomarker in any American or European guideline.

A possible study limitation is the absence of data on concomitant nocturnal oximetry, ABGs, and MIP/MEP tests, which could allow to confirm and further evaluate the relationship between serum chloride and early respiratory failure. This limitation is due to the retrospective nature of our study and needs to be addressed in future prospective studies. Moreover, we were not able to evaluate serum chloride values during disease progression due to the lack of longitudinal data systematically collected.

Nevertheless, our data suggest that serum chloride evaluation should be included as an effective and low-cost marker of hidden or impending respiratory failure in ALS patients. Its wide availability and operator independence confirmed its potential role both in spinal and bulbar onset patients, being able to provide screening information that could guide more detailed respiratory function tests.

## Data availability statement

The raw data supporting the conclusions of this article will be made available by the authors, without undue reservation.

## Ethics statement

The studies involving human participants were reviewed and approved by Ethical Committee of the Azienda Ospedaliero-Universitaria Città della Salute (Prot. N. 0036344). The patients/participants provided their written informed consent to participate in this study.

## Author contributions

UM and MG contributed to conception and design of the study and wrote the first draft of the manuscript. UM, MG, EM, AB, RV, MT, and PC organized the database. UM performed the statistical analysis. CM and ACan wrote sections of the manuscript. ACh and ACal revised the manuscript critically for important intellectual content. All authors contributed to manuscript revision, read, and approved the submitted version.

## Funding

This study was in part supported by the Italian Ministry of Health (Ministero della Salute, Ricerca Sanitaria Finalizzata, grant RF-2016-02362405), the European Commission’s Health Seventh Framework Programme (FP7/2007–2013 under grant agreement 259867), the Italian Ministry of Education, University and Research (Progetti di Ricerca di Rilevante Interesse Nazionale, PRIN, grant 2017SNW5MB), the Joint Programme—Neurodegenerative Disease Research (Strength and Brain-Mend projects), granted by Italian Ministry of Education, University and Research, the European Union’s Horizon 2020 Research and Innovation Programme (Brainteaser Project, grant GA101017598). This study was performed under the Department of Excellence grant of the Italian Ministry of Education, University and Research to the “Rita Levi Montalcini” Department of Neuroscience, University of Torino, Italy.

## Conflict of interest

ACal received research grant from Cytokinetics. ACh served on scientific advisory boards for Mitsubishi Tanabe, Roche, Biogen, Denali Pharma, AC Immune, Biogen, Lilly, and Cytokinetics. The sponsor organizations had no role in data collection and analysis and did not participate to writing and approving the manuscript. The information reported in the manuscript has never been reported elsewhere.

The remaining authors declare that the research was conducted in the absence of any commercial or financial relationships that could be construed as a potential conflict of interest.

## Publisher’s note

All claims expressed in this article are solely those of the authors and do not necessarily represent those of their affiliated organizations, or those of the publisher, the editors and the reviewers. Any product that may be evaluated in this article, or claim that may be made by its manufacturer, is not guaranteed or endorsed by the publisher.
